# Nurses Navigating Mental Health During Uncharted Times: Self, Others, Systems (S.O.S)!

**DOI:** 10.1177/08445621241266291

**Published:** 2024-07-26

**Authors:** Chaman Akoo, Sheri Price, Kimberly McMillan, Kenchera Ingraham, Abby Ayoub, Shamel Rolle Sands, Mylène Shankland, Ivy Bourgeault

**Affiliations:** 1 School of Nursing, 6363 University of Ottawa, Ottawa, Canada; 2 School of Nursing, 3688 Dalhousie University, Nova Scotia, Canada; 3 School of Nursing, 3158 University of Alberta, Edmonton, Canada; 4 Institute of Feminist and Gender Studies, 6363 University of Ottawa, Ottawa, Canada; 5 School of Sociological and Anthropological Studies, 6363 University of Ottawa, Ottawa, Canada

**Keywords:** Covid-19, nursing, burnout, mental health, qualitative research, Canada

## Abstract

**Study Background:**

The nursing profession is facing a multiplicity of stressors that have both predated and been exacerbated by the Covid-19 pandemic. The emotional and physical demands entailed in nursing predispose nurses to suboptimal mental health and burnout.

**Purpose:**

This paper draws upon the narrative interviews of 53 Canadian nurses as part of a larger pan-Canadian, cross disciplinary study that examined the gendered experiences of mental health, leaves of absence, and return to work of 7 professions.

**Methods:**

Thorne's interpretive descriptive guided Iterative and thematic analysis which identified three predominant themes within the nursing dataset, this paper focuses on the substantive theme of ‘*Navigating it Alone,*’

**Results:**

Nurses expressed a profound sense of isolation at 3 particular levels: at home, at work, and in systems – while simultaneously balancing uniquely gendered familial responsibilities and workplace demands.

**Conclusions:**

These results illuminate instrumental pathways for stakeholders to attenuate the personal and professional pressures that continue to be disproportionately carried by nurses as they navigate these particularly challenging times.

## Background and purpose

The Covid-19 pandemic has been unprecedented in scale, having significant burdens falling disproportionately and inequitably on frontline healthcare providers (Manzano García & Ayala Calvo, 2021). Multiple waves of Covid-19 spurred by variably virulent variants have provided minimal recuperative time for nurses and the crisis has further exacerbated long-existing professional inequalities such as comparatively low compensation, inadequate working conditions, chronic staff vacancies, and increased physical, emotional, and moral injuries (Akoo et al., 2024; Stelnicki et al., 2020). Covid-19 continues interminably as an existential threat worldwide, and healthcare systems continue to reconcile the ongoing challenges in the nursing workforce (Akoo et al., 2024; Bourgeault et al., 2022).

### Increased workplace demands

Nursing personnel play a pivotal role in the provision of care; care which, amidst increasingly unpredictable and uncertain work environments, is coming at increasing personal cost (Li et al., 2022). Studies have identified nursing as one of the most stressful professions, and the pandemic has further amplified anxiety, depression, post-traumatic stress disorder (PTSD), and psychological distress among clinicians (Ashley et al., 2021; Li et al., 2022; García-Vivar et al., 2023). Researchers have identified that these psychological challenges often result in staff absenteeism and leaves of absences (LOA) (Akoo et al., 2024). In addition to navigating pre-existing workplace challenges, nurses have identified new stressors such as insufficient or poorly fitting personal protective equipment (PPE), navigating redeployment, enforcing visitor restriction policies, and keeping abreast rapid, and at times – contradictory - policies and clinical procedures (Akoo et al., 2024; Fernández-Basanta et al., 2022; Li et al., 2022). Such cumulative stressors during the pandemic have resulted in what Fernández-Basanta and colleagues (2022) metaphorically represented as a “backpack” that nurses carry as they navigate the weight of “emotional and physical demands of care, uncertainty, social rejection, and nonrecognition of the institution” (p. 4). The resulting burnout and mental health challenges have direct implications for patient safety and organizational outcomes (Stelnicki et al., 2020).

### Gendered nature of burnout

The existing body of research largely considers burnout as gender neutral, and therefore, lacks a gender based analysis (Aldossari & Chaudhry, 2021). This is surprising and problematic provided the significant differences between the gendered experiences of work and the inequitable distribution of household duties and childcaring responsibilities (Smith et al., 2022). What is recognized however, is the concomitant pressures of work and caregiving responsibilities generate immense pressure (Dall’Ora et al., 2020). Therefore, it is necessary to better understand the gendered perception and contributing factors of adverse mental health, burnout, and absenteeism, particularly as these pressures have escalated to unsustainable levels.

Globally, nursing remains a predominantly female profession (World Health Organization [WHO], 2020), and pandemics have historically exacerbated structural gender inequities by further intensifying pre-existing asymmetrical divisions of household labor (Johnston et al., 2020). A recent provincial report in Canada highlighted that 48.3% of frontline nurses reported their workload *significantly* increased during the pandemic, and in concert to increased work related demands, 79.8% of respondents also disclosed increased familial caregiving responsibilities (Registered Nurses Association of Ontario [RNAO], 2022).

It is imperative to elucidate the contributors of stress and burnout from a gender lens, to construct appropriate supports and retain highly knowledgeable professionals, arguably, when their skills and knowledge have never been so in demand. The egregious omission of a gender lens contributes to a lack of gender targeted policy measures and interventions. The purpose of this study was to examine the mental health, leave of absence and return to work experiences of seven professional case studies, coincidentally during the Covid-19 pandemic. This paper will report specifically on the findings of the nursing case study and although the study took place during the pandemic, the participants’ experiences are not solely specific to this timeframe.

## Methods and procedures

### Design

The study was driven theoretically by intersectionality (Hankivsky et al., 2010), with a focus on the gendered lived experiences of participants in different personal, family and work contexts, as well as an examination of how lived experience is mediated by professional work culture. This research drew upon Acker's (1990) approach to gendering work in organizations. Acker (1990) contends that work structures within organizations are not gender neutral and the assumption of a “disembodied, universal worker” serves to “marginalize women's experiences.” Recognizing the embodied nature of the work allows a more comprehensive understanding of the gendered nature of work in organizations and how work can have different meanings for different genders (Leidner, 1991).

Each case study utilized a multi-method approach composed of document analysis, stakeholder interviews, worker surveys, and worker interviews. In this investigation, the nursing researcher team employed a descriptive, qualitative methodology (Thorne, 2016), to better understand the experiences of frontline nursing professionals and key stakeholders as it pertains to nurses emotional well-being, barriers and supports to mental health, leaves of absence, and return to work. This paper focuses exclusively on the substantive qualitative results from the nursing case study which focused on understanding the mental health, leave of absence and return to work experiences of frontline Canadian nurses.

### Qualitative study sample

A sample of fifty-three nurses from Canada were invited to participate in semi-structured, one-on-one interviews, purposively selected from survey respondent volunteers to represent a range of nursing workforce experiences. In this sample, nurses from the following subclassifications were included: 34 Registered Nurses (RNs), 6 licensed Practical Nurses (LPNs), 1 Psychiatric Registered Nurse (RPN), 7 Nurse Practitioners (NPs), and 5 Undisclosed. This sample included 46 female, 7 male, and 0 non-binary or gender fluid individuals. Fourteen participants reported caregiving responsibilities outside of work to either children or family members, and 38 participants reported having taken a leave of absence during their career and it was not identified if this occurred solely during the Covid-19 pandemic. The sample was inclusive of those who chose not to return to their workplace and sought alternative nursing employment. Representation from all provinces and territories were sought, however no participants volunteered from the three northern Canadian territories.

### Data collection

Two team members conducted virtual interviews in English or French, which took place between September 2020 and July 2021, and were audio recorded for analysis. Interviews were approximately one hour in length, and the questions remained the same throughout data collection, following a semi-structured interview guide. One researcher led the interview, while the other concentrated on taking notes. The interview questions focused on demographics, mental health, leaves of absence, and return to work experiences (See Table 1). Interview audio files were subsequently transcribed by Otter.ai software and reviewed and verified for accuracy by members of the nursing research team prior to data analysis.

**Table 1.** Sample of interview questions.

Reflecting on your family characteristics and circumstances, what would you describe as most rewarding? Most challenging?
How would you describe your workplace culture?
We’re curious about whether you think gender (and other social identities) affects one's mental health experiences at work.
Have you ever taken a leave of absence from work for mental health reasons?
What changes could have made things better for you at work?

### Ethics

Research Ethics Board approval was obtained from the the University of Ottawa and fourteen additional institutional review boards due to the size and scope of the parent study. Before interviews, consent forms were emailed to eligible participants and informed consent was obtained. All research team members have experience conducting qualitative interviews on potentially distressing and emotive topics, and caution was exercised to conduct interviews in a sensitive manner. All data were de-identified, anonymized, and participants were issued an alphanumeric code. All transcriptions were uploaded to the Secure Empirical Analysis Lab (SEAL) – and access was restricted to the core analysis team.

### Data analysis

The qualitative component of this study utilized interpretive description (ID) (Thorne, 2016), which was selected for its methodological flexibility and support for various data collection and analytical processes. An inductive approach to analysis allowed the researchers to explicate meaningful themes and patterns related to the phenomena of interest (Thorne, 2016) through reflective examination and questioing of the data in order to produce rich, meaningful findings (Thompson Burdine et al., 2021; Thorne et al., 2004). Data analysis commenced with reading each transcript, making note of significant words and phrases, as well as overall participant elocution. Thematic analysis ensued, which is an established method to identify, analyze, and interpret patterns (Braun & Clarke, 2006). Thematic analysis allows the theorizing of “sociocultural contexts, and structural conditions, that enable the individual accounts” (Braun & Clarke, 2006, p. 14). Analysis occurred sequentially, with each researcher independently analyzing the data and meeting for consensus on emerging patterns and themes.

### Trustworthiness and credibility

Credibility was established by adhering to the criteria articulated by Thorne (2016) consisting of epistemological integrity, representative credibility, analytic logic, and interpretive authority. Representative credibility refers to application of a sampling strategy that is reflective of the phenomena under study (Thorne, 2016), which was achieved through a diverse sample of participants. Representative credibility was also enhanced through prolonged engagement with data by multiple members of the research team with qualitative research expertise. Epistemological integrity refers to a “defensible line of reasoning” which conveys epistemological coherence from the research question to data sources and research findings (Thorne, 2016, p. 233). This was demonstrated through decisional strategies that respect the epistemological position of constructivism in the research process. Analytic logic was supported through the creation of an audit trail and explicit reasoning pathway (Thorne, 2016). This was also supported through grounding themes in rich, verbatim language. Interpretive authority respects that all knowledge is subjective and contextual, and as such, to provide assurance of the trustworthiness of findings the researchers made explicit their own biases and assumptions (Thorne, 2016). Researchers maintained reflexive journals throughout the study. This ensured that the researchers were conscious of, and carefully examined how, personal opinions, beliefs, and biases could influence the outcome of the research. These four criteria served as our compass when determining when to cease data collection. We analyzed data as it was collected, and once we believed we understood the data in ways that upheld these notions outlined by Thorne (2016), we stopped collecting data. Both Thorne (2020) and Braun and Clarke (2021), who collectively shaped our methodology, take issue with the concept of saturation, therefore the concept was not used to guide when data collected would cease.

## Results

Findings elucidated through an intersectional gender analysis centered on the theme “*Navigating it alone,*” recognizing that gender roles and norms mediate the influence of both personal experiences and health systems (Smith et al., 2022). Norms are implicit and embedded within institutions constraining whose contributions are recognized and valued, and whose needs are accommodated and supported (Morgan et al., 2016). As such, this analysis centers how different gendered roles and expectations are experienced implicitly. The following analysis recognizes that (health) systems are gendered, and these findings seek to center nurses’ narrative accounts within these larger structures of gender-based inequity. It is of significance to note that data were collected during the Covid-19 pandemic, reflecting a unique space and time in our collective history and serving as the context for participant experiences.

### Navigating it alone at work

A considerable number of participants spoke candidly about the isolation they experienced as they navigated workplace challenges and their ensuing emotional burdens. Burdens which, have only multiplied as nurses have shouldered exceptional levels of personal and professional uncertainty and loss. One participant, with an extensive and varied career, spoke unreservedly about the cumulative traumas that occurred during her career as a registered nurse. She described navigating these emotional traumas in isolation, without any institutional recognition, until eventually, she could no longer manage:

… I find in general…there's very little support and understanding to exactly the emotional toll that it [nursing work] can take. I've had some very traumatic experiences– they [employer] just not understanding that…when you've just told someone their loved one has been killed in a car accident. And you have to walk into the next room and continue nursing on another patient and not react. It just… it drowns you eventually**.** (Participant 51, Registered Nurse – Acute Care, Manitoba)

She shared that these cumulative career traumas predated the pandemic, however, for her they culminated into a significant mental health crisis when she experienced the death of her father during the pandemic. Throughout it all, she noted how alone she felt, feeling reluctant to share her struggles for fear of workplace repercussion and stigma. Eventually, she sought professional care and recognized the importance of reaching out and finding supports. She eloquently states:

We're a caring profession but when we need somebody to take care of us, it's really hard to find. We have to do it ourselves. (Participant 51, Registered Nurse – Acute Care, Manitoba)

One new graduate nurse spoke to a lack of support in the workplace - where she often felt unsupported when dealing with the aftermath of critical events. She spoke to the lack of formal debriefing, or even acknowledgement from colleagues about how emotionally taxing managing critically ill [pediatric] patients can be:

But I think what was really needed was that formal recognition of that's scary, like that was a life at danger, and how are we going to respond to that, but instead it was kind of seen as it's part of the job. I guess it happens. And I think that really weighed down my own mental health and emotional health being there as well. (Participant 1, Registered Nurse – Acute Care, Ontario)

She articulated that it doesn’t need to be this way. That even within fast-paced, high acuity environments nurses can thrive, but these environments need to be equipped with meaningful supports for clinicians to do so. Supports which, for her, were not in place:

I think nurses can still thrive in those environments with the right support. But I think that support really needed to be there. And it wasn't. (Participant 1, Registered Nurse – Acute Care, Ontario)

Many participants explicitly voiced a lack of support from administration and institutions, and particularly relating to the Covid-19 pandemic, participants commented on the superficial and performative support provided by their managers and administrators. One veteran emergency room nurse spoke to this absence of institutional support, while navigating his own mental health deterioration and that of his colleagues. He illustrated how he increasingly bears witness to colleagues breaking down and crying at work due to the workload, workplace violence, and short staffing. He felt that he and his colleagues were largely navigating systemic workplace challenges alone and when describing management support as “minimal,” he stated:

They like to send emails, which I often can't get to throughout the course of my shift. And the emails have some nice platitudes in them. ‘We're all in this together’ and all this fun stuff, but then […] When things go sideways, they're not actually there with us. (Participant 25, Registered Nurse – Acute Care, Alberta)

Participants voiced that in addition to navigating workload and workplace traumas without institutional support, once they sought assistance and realized they needed to take a leave of absence, they found that once again, they were navigating this process alone. The following participant sought help through her Employee Assistance Program (EAP) but found the experience disconcerting and unprofessional - note how her assigned therapist offloaded the responsibility of supporting her because she was a “professional”:

I started out by contacting my EAP and through my EAP, I was set up with a […] counselor and it was a very weird setup… the person just recommended a bunch of books and said, ‘Well, you're a professional, and you know what you need to do so…,’ which was not helpful at all. (Participant 28, Nurse Practitioner – Community, Nova Scotia)

Another participant vividly illustrated her experience filling out the forms needed to process her leave of absence, and how there was an absence of support navigating paperwork, which was particularly challenging while she was experiencing an acute mental health crisis:

*…And then basically, I navigated it on my own…severe depression, suicidal and filling out online forms; I mean, it's ridiculous. It's just a good thing that I worked in workers compensation for 14 years, so I could just kind of shut things down and just go, you just got to go through the process […] Just fill out the forms. Do your thing. Don't react. Just get it done. (Participant 29, Registered Nurse – Community, British Columbia)*

In addition to feeling as though they were navigating it alone at work, participants also described navigating it alone in their lives outside of nursing work.

### Navigating it alone at home

Nurses expressed disproportionate caregiving responsibilities, both preceding and notably amplified during the Covid-19 pandemic. The Covid-19 pandemic affected not only the nurses themselves, but also members of their family – which had implications in the context of caregiving responsibilities in the home. Nurses described the additional pressure of having to navigate their family's well-being alongside their own mental health, and the toll this had on their ability to cope. Some participants explicitly highlighted challenges relating to the unequitable responsibility of coordinating childcare. The following nurse described how the pandemic resulted in her working from home, but that this domestic proximity also positioned her to be disproportionately responsible for childcare. The following vignette speaks to this challenge, as well as the emotional labor she undertook to convince her husband that she needed more support:

My husband continued to work outside of the home, and I worked inside the home. And so again, it became my primary responsibility to figure out how to make that work. How to care for the kids and continue to do my job. And I do have a very understanding and supportive husband, but then it was on my onus to have to describe to him, or convince, or help him understand, why we then needed someone to come help take care of the kids for some periods of time, because it wasn't possible for me to be meeting their demands, as well as working. (Participant 7, Registered Nurse – Community, Ontario)

Another participant spoke of similar challenges with caregiving but explicitly highlighted that this asymmetrical division of labor existed well before the pandemic. She illustrated her husbands’ default expectation that she would use her sick time to attend to their children, resulting in increased work absenteeism. She recounts how this implicit caregiving expectation resulted in excessive sick time utilization, and congruent with the previous participant, how she had to expend emotional energy trying to convince her husband to equitably share this division of labor:

*In terms of that [childcare] my husband wasn't very supportive. He just felt like ‘Oh well, you know, you have sick time […] you go get the kids.’ He just always thought that, ‘Oh, you could do it.’ […] I remember just having arguments with him about ‘Can't you take the day off?’ Like, I can't keep taking all this time off work. (Participant 38, Registered Nurse - Community, British Columbia)*

Unequitable caring responsibilities also involved extended family members. One participant shared that despite not having her own children, she became the default caregiver for an extended family member before the pandemic:

I took a two week leave of absence in my current job. I had an uncle who became gravely ill. He was a bachelor. He had no children. There was a lot of other issues going on. He was a hoarder. He was bankrupt. And there was nobody else to help him. […] So, I ended up being in charge of him and it was very, very stressful, especially in the beginning, when so much had to be done. I took a two week leave of absence. (Participant 46, Registered Nurse – Acute Care, Manitoba)

She further reflected on this gendered caregiving experience and the resulting stress:

They [family] leaned on me quite heavily. And I think, that's part of being female. That's part of being a nurse. And it was very, very stressful. (Participant 46, Registered Nurse – Acute Care, Manitoba)

The data further highlights the high level of emotion labor involved in this caregiving work in the home, and the toll it took on the emotional well-being of some participants, while coupled with the emotionally demanding nature of their nursing work outside of the home. Challenges relating to navigating guilt were also evident in some accounts, with some nurses questioning if they should reduce their hours of paid work or take a leave of absence, to attend to unpaid work in the home. This is revealed in the following narrative:

*That is a challenge, definitely with Covid, I would say. At times, it can be an issue because sometimes what you're seeing at work is so close to you that you think that this may happen to your own family members at home, especially with Covid. My daughter goes to one of the largest daycares in the southeast corner of this province, so, as I see testing numbers of children coming in, I'm worrying is my daughter going to be the next one? So then you have guilt, should I cut back [working hours], stay at home with her, so she doesn't have to go to childcare and maybe get Covid? (Participant 40, Registered Nurse – Community, Saskatchewan)*

Another participant outlined the highly interdependent nature of her ability to work as a nurse while also being the primary support for her husband's mental health. Consequently, she felt obligated to take time away from work when her husband was struggling with PTSD. She reflected during our conversation that she felt her nursing career was constrained to some degree because she had to attend to her husband and children. She explained:

But the one of the reasons I gave up my job is because my husband worked for the [police] and he retired, but he had to retire early because he developed PTSD. And so, when Covid happened, I worked for the college. And in September, everything went online. And I found it too difficult to learn all the technology required. But in addition to that, it was really impossible for me to turn our house into a live classroom six hours a day when my husband was not well, mentally. So that was the reason I had to give up my job. (Participant 52, Registered Nurse – Community, Newfoundland)

Gender did explicitly shape this particular subtheme – male participants did not report the same level of care-related expectations and responsibilities at home. In addition to navigating it alone at work and home, participants also felt they were unsupported by broader systems, both with and beyond the constructs of their professional lives.

### Navigating it alone in systems

Participants recognized themselves as embedded in larger communities and voiced that they felt abandoned and unsupported by professional unions, regulatory bodies, and even some provincial governments. They articulated that despite the amplified rhetoric of calling nurses “healthcare heroes” during the pandemic many did not feel supported by systems or societies that appeared to devalue nursing. This was most evident when participants spoke of inadequate compensation and pay equity, which participants linked nursing being a profession where women predominate. One participant noted how nurses are “oppressed” and “not prioritized in healthcare” (Participant 24, Registered Nurse). Another participant decidedly directed her anger at provincial governments for contributing to poor nursing mental health and attrition:

*I mean, especially at this time when I know so many nurse practitioners that are wanting to leave the industry - get out of the profession in a heartbeat - and then all the nurses that I work with they're all like, ‘I'm rethinking why I'm here.’ …[premier] passing Bill 124…he says you as nurses are not allowed to have more than a 1% pay raise for the next five years. That effects your mental health because you don't feel appreciated. Sure, we're healthcare heroes. I've put myself on the frontline for the last year and a half. I've been exposed to Covid a couple of times at work - no pay, I get no pay. (Participant 43, Nurse Practitioner – Community, Ontario)*

Another nurse from a different Canadian province living under a comparable governmental ethos of austerity described a similar sentiment and expressed provincial government attitudes towards healthcare - and by extension nurses - as “unsupportive,” “oppressive,” and “punitive.” She further explained how this lack of government support has influenced her as the sole breadwinner in her household, and how in order to supplement her income she has had to open an ‘Airbnb’ in her home. She noted this pressure to always maintain her household to support her business leaves her little time to decompress and relax:

*What would also make my personal life better is if we had a contract with the government. We haven't had a raise in five years. It's had a financial impact. I've had to start a small business. I have an Airbnb business in my home in order to supplement the loss of income I've experienced from no pay raises and in a long, long time.* (Participant 46, Registered Nurse – Acute Care, Manitoba)

While provincial governments had a considerable influence on the lack of support nurses experienced, some participants also voiced a lack of guidance from professional unions. One participant in particular spoke at length about the toxic management of her workplace and the ensuing attrition. She explained that many individuals felt they had no choice but to leave their workplace or take a leave of absence, since they had no alternative course of action through their union:

The union, according to our contract is not allowed to help with workplace harassment, they can walk with you, but they can't fight for you. (Participant 21, Registered Nurse – Community, New Brunswick)

In concert with this, another participant, a nurse practitioner in the community who held many different roles, also shared how she and her colleagues felt abandoned and unsupported by regulators and unions:

So majority of your [nurse] practitioners are not unionized unless you're hospital based. So there was no union to fall back on. The [Nurse Practitioner Association], they advocate for nurse practitioners [collectively]. They don't advocate individually. So unfortunately for a lot of nurse practitioners, you are fighting your own battle, there's nothing else. Nobody else to support you. (Participant 43, Nurse Practitioner – Community, Ontario)

Participants in this study overwhelmingly shared the ways in which they navigated a variety of complexities at work, home, and at a systems level, feeling largely isolated and unsupported. Because of this experience, they held considerable insight into how to rectify and attenuate such demands on nurses, and nursing, as noted in the findings.

## Discussion

As evident within the narratives of these study participants, the increased demands and deterioration of workplace conditions have amplified the physical and emotional strain on nurses. Nurses in our study emphasized how they feel both unsupported in being able to care for their patients, and alone in being able to navigate their own mental health and well-being. There has been a lot of emphasis in the literature on the need for nurses to demonstrate resiliency and much attention on self-care strategies (Udod, MacPhee & Baxter, 2021). However, this discourse of nurse resiliency can reinforce the harmful belief that mental health is solely an ‘individual’ issue thereby disregarding the need for supports at the organization and system levels. Nurses not only need support to address workplace conditions but also need assistance in terms of managing their mental health. Expecting nurses to be more resilient is not an effective solution to the multitude of challenges they face in the workplace. Several scholars have recognized the limits to individual resilience (Mahdiani & Ungar, 2021; Scrine, 2021) and have advocated for healthcare leaders to foster organizational resilience – which entails ensuring workplace structures and human resource supports are put in place to support employees (Rangachari & Woods, 2020).

Our findings also further elucidate the gendered nature of nurses’ mental health experiences. Gender inequity in the division of labor in the home is well established (Johnston et al., 2020; RNAO, 2022) but by way of the pandemic, these inequities require further attention. The pandemic has shone a light on the inequitable burdens placed on women both at work and at home – notedly as it pertains to caregiving responsibilities (Morgan et al., 2022). Examples include: navigating rapidly changing child education scenarios; with school closures, virtual classrooms, and varying return to in-person teaching policies (Carli, 2020; Power, 2020). Nurses (who are predominately women) were also disproportionately responsibility for caregiving in the home during the pandemic (Morgan et al., 2022). As a result of these additional pressures placed disproportionately on women, research has demonstrated that female nurses faired much worse than their male counterparts in terms of work-related stress during the pandemic (Morgan et al., 2022; Zhang et al., 2020). One review by Zhang et al. (2020) found female nurses reported the highest levels of burnout, anxiety and depressive symptoms during the pandemic compared to other healthcare professionals. Moreover, a systematic review and meta-analysis by Sun et al. (2020) on the psychological impact of Covid-19 on healthcare professionals found that women working at the frontline in healthcare experienced the highest incidence of mental health concerns, specifically anxiety and depression. There is a dire need to ensure that we design interventions specifically to address the burdens experienced by women to keep them in the workplace with minimal impacts to their mental health. Some strategies to mitigate the gendered impact of crisis on women healthcare professionals include childcare and assistance with dependent's caregiving, economic support and supporting work absences (Morgan et al., 2022). Moreover, women need to be able to voice their concerns (and those concerns must be heard) and may need assistance in identifying their specific needs to ensure that ensuing strategies and interventions are targeted to meet their needs.

Ultimately, calls to action must move beyond individual level interventions and incite organizational and system level empowerment. As identified in our study, there is a great need for interventions at the unit and organizational levels (Chatzittofis et al., 2021; Rieckert et al., 2021). Moreover, these interventions should involve shared decision-making with input from nurses and other front-line health care providers to create a culture of support that directly meets their needs (Cho et al., 2021). Creating a caring environment and supportive workplace relationships requires strong leadership and a culture of compassion (Lecca et al., 2020). Recent research exploring mental health leave of absence and return to work among nurses across Canada during Covid-19 highlighted the significance of compassionate acts in the workplace, whereby even small acts of compassion supported the mental health and wellbeing of nurses, even for those on mental health leave and those returning to work following a leave (Akoo et al., 2024). This finding suggests that fostering compassionate communities within the healthcare workforce may serve as a starting point for supporting and retaining nurses. Due to the various Covid-19 related traumas and associated grief/loss nurses have experienced, which contributes to mental health decline and attrition, the fostering of compassionate communities within the healthcare workforce is timely, relevant and necessary. Although traditionally associated with palliative care provision, the concept of compassionate communities (Kellehear, 2005; Vanderstichelen et al., 2022) should not be overlooked as an innovative approach to repairing the nursing workforce.

Organizational factors such as the culture of the work environment, communication, and support from supervisors/management, are well recognized in the literature as significant influences on the mental health symptoms of health professionals (Zhang et al., 2020). Supportive work environments and the cultivation of open and transparent communication between administrators and employees is essential (Shah et al., 2021). Enhancing social support provided by colleagues and managers is critical to ensuring a positive workplace culture and has been proven to counter the effects of work-related stress (Lecca et al., 2020). Having the opportunity to discuss challenges and concerns openly and safely, including struggles with mental health have been repeatedly identified by nurses as helpful. Creating psychologically safe environments, where employees can openly express thoughts without fear of stigma and repercussion are essential. Strategies such as team huddles and debriefing opportunities are also noted to be supportive mitigation strategies (Haugland et al., 2023). Solutions focused on a systems-level would entail the inclusion of the uniquely gendered voice of nurses at key decision-making tables regarding pandemic response and recovery. Globally, many gender-focused organizations, like Women in Global Health (2023), have raised the issue of the lack of women in key decision-making roles. Given the predominance of women in nursing, this overlaps with the lack of representation of nursing at system-level tables. Nurses would bring their unique health system experiences and perspectives as well as the under-recognized gender role within families toto the fore the intersecting challenges they other face navigating it alone at work and at home.

## Conclusion

Findings from this study suggest that gender plays a significant role in how nurses experience and must navigate their mental health and well-being both in their professional and personal lives, often left to do so in solitude. As such, nurses could benefit from, and contribute to system level structural changes that would more effectively acknowledge the positionality of women as it relates to both paid and unpaid caring work. It is imperative that a gendered lens be utilized when addressing the current health human resource crisis, notably within the nursing profession, in hopes of repairing the nursing workforce in ways that creates a stable workforce where nurses feel supported in their work, which at times is incredibly mentally and emotionally taxing.
